# Prostate Artery Embolization for Complete Urinary Outflow Obstruction Due to Benign Prostatic Hypertrophy

**DOI:** 10.1007/s00270-016-1502-3

**Published:** 2016-11-16

**Authors:** Simon Chun Ho Yu, Carmen Chi Min Cho, Esther Hiu Yee Hung, Peter Ka Fung Chiu, Chi Hang Yee, Chi Fai Ng

**Affiliations:** 1 Department of Imaging and Interventional Radiology, The Chinese University of Hong Kong, c/o Rm 2A061, 2/F, Main Clinical Block and Trauma Centre, Prince of Wales Hospital, 30-32 Ngan Shing Street, Shatin, New Territories, Hong Kong SAR; 2 Vascular and Interventional Radiology Foundation Clinical Science Center, The Chinese University of Hong Kong, Shatin, Hong Kong SAR; 3 Department of Surgery, Prince of Wales Hospital, The Chinese University of Hong Kong, Shatin, Hong Kong SAR

**Keywords:** Benign prostatic hypertrophy, Acute retention of urine, Prostate artery, Embolization

## Abstract

**Background:**

We aimed to evaluate the effectiveness of PAE in weaning of catheter and relieving obstructive urinary symptoms in patients with acute urinary retention (AUR) due to benign prostatic hypertrophy (BPH) and failed trial without catheter (TWOC).

**Materials and Methods:**

In this prospective study approved by the institutional review board, a signed informed consent was obtained. Eighteen consecutive patients with AUR due to BPH and failed TWOC were recruited. Nineteen consecutive patients with BPH but without AUR were recruited as a control. Patients with CTA evidence of arterial occlusion or significant stenosis along the prostate artery access path were excluded. PAE was performed using microspheres (100–300 μm diameter). Outcome assessment included successful weaning of catheter in 2 weeks, procedure-related complications, change of symptomatology and urodynamic findings at 1 month as compared to baseline, percent non-perfused prostate volume, and prostate volume reduction on MRI at 2 weeks.

**Results:**

Two patients in the study group and four in the control group were excluded due to arterial pathology. Embolization of bilateral prostate arteries was achieved in all patients in both the groups (100%). There was no complication. The catheter was successfully weaned in 87.5% (14/16) of patients within 14 days in the treatment group. There was no significant difference in patient demographics, prostate characteristics, and all outcome assessment parameters between both the groups.

**Conclusions:**

PAE was probably safe and effective in weaning of catheter and relieving obstructive urinary symptoms in patients due to BPH, with treatment outcomes comparable to those without AUR.

## Introduction

The standard initial management of acute urinary retention (AUR) due to benign prostatic hypertrophy (BPH) is immediate bladder catheterization followed by trial without catheterization (TWOC) after 3 days of catheterization [1]. Alpha-blockers are playing an important role in managing patients with symptomatic BPH. In patients presenting with a first episode of spontaneous AUR related to BPH, alpha-blocker such as Alfuzosin XL 10 mg per oral daily significantly increases the success rate of TWOC [2, 3]. Patients with BPH-related AUR and failed TWOC while on alpha-blocker are usually treated with transurethral resection of prostate (TURP), which is effective in relieving the complete obstruction at the bladder outlet. However, TURP is associated with a number of complications such as blood loss requiring blood transfusion (0.4–7%), urinary incontinence (30–40%), retrograde ejaculation (65–80%), impotence (5%), infection, urethral stricture, and need for surgical retreatment for lower urinary tract symptoms (3–14.5%) [4–7]. Others include infection and urethral stricture. Prostate artery embolization (PAE) has been introduced as a treatment for patients with symptomatic BPH [8–12]. Although PAE is generally less invasive than TURP, the effectiveness of PAE in relieving the obstructive effect of BPH in patients with AUR and failed TWOC is unclear. We aim to evaluate the effectiveness of PAE in the weaning of catheter and relieving obstructive urinary symptoms in patients with AUR due to BPH and failed TWOC.

## Materials and Methods

### Study Design

This was a prospective study that was conducted in accordance to the Declaration of Helsinki and international standards of Good Clinical Practice, and approved by the institutional review board. A signed informed consent was obtained from all patients. It was hypothesized that for patients with a Foley catheter placed for AUR due to BPH and failed TWOC despite alpha-blocker therapy, PAE is effective in the weaning of catheter and relieving the obstructive urinary symptoms to a degree comparable to that in patients with symptomatic BPH without AUR. From June 2015 to March 2016, 37 consecutive patients who had fulfilled all the eligibility criteria were recruited into the study (Table 1). Eighteen patients with AUR due to BPH and failed TWOC despite having on alpha-blocker (Alfuzosin XL 10 mg oral daily) were allocated into a study group. These patients were on a waiting list for TURP and had an indwelling Foley’s catheter placed for 21–107 days (average 69.5 ± 32.8 days) before PAE. Nineteen patients with symptomatic BPH but without AUR, being on alpha-blocker (Alfuzosin XL 10 mg oral daily), with International Prostate Symptom Score (IPSS) ≥ 15, quality of life score 3 or above, and urine peak flow rate less than 15 mL/s, were allocated into a control group. The patients were then assessed with CTA for evidence of vascular occlusion or severe stenosis along the relevant vascular access path. Two patients in the study group were excluded due to CTA evidence of complete occlusion of one of the internal iliac arteries, or severe stenosis at the origin of one of the inferior vesicle arteries. Four patients in the control group were excluded because of severe stenosis of one of the inferior vesical arteries. Sixteen patients in the study group and 15 patients in the control group received PAE (Table 2).

**Table 1:** Eligibility criteria

Inclusion criteria	Exclusion criteria
1. Willing to sign an informed consent	1. Active urinary tract infection, or
2. Age between 50 and 80 years old, and	2. Biopsy proven prostate or bladder cancer, or any cancer other than basal or squamous cell skin cancer, or
3. Known history of symptoms of lower urinary tract obstruction attributable to benign prostatic hypertrophy, and	3. Bladder atonia, neurogenic bladder disorder or other neurological disorder that is impacting bladder function, or
4. Prostate size ≥50 g on ultrasound, and	4. Urethral stricture, bladder neck contracture, sphincter abnormalities, urinary obstruction due to causes other than BPH, or other potentially confounding bladder or urethral disease or condition, or
5. Serum PSA level <4 ng/mL or ≥4 with cancer excluded by biopsy, unless biopsy is refused by the patient	5. Previous surgery or transurethral resection of prostate, needle ablation, balloon dilation, stent implantation, or any other invasive treatment to the prostate, or
For patients in the study group	6. Patient unable to receive MRI imaging, or
1. Presented with acute urinary retention, and	7. Baseline serum creatinine level >1.8 mg/dl, or
2. Managed with transurethral placement of Foley catheter in urinary bladder, and	8. Known upper tract renal disease, or
3. Put on Alfuzosin XL 10 mg oral daily, and	9. Active prostatitis, or
4. Failed trial without catheter	10. Previous rectal surgery other than hemorrhoidectomy, or history of rectal disease, or
For patients in the control group	11. History of pelvic irradiation or radical pelvic surgery, or known major iliac arterial occlusive disease
1. Quality of life score ≥3, and	
2. Urine flow rate <15 mL/s at a total bladder volume ≥150 mL, and	
3. Prostate size of at least 50 grams as measured on ultrasound, and	
4. On Alfuzosin XL 10 mg oral daily

**Table 2:** Patient demographics and baseline characteristics

Baseline characteristics	Study group (*n* = 16)	Control group (*n* = 15)	*P* value
Age (year)	66 (60.3, 70.3)	66 (60, 72)	0.953
Prostate volume (mL)	77 (55.1, 94.3)	65.6 (41.6, 81.9)	0.165
Serum PSA level (μg/L)	14 (7.5, 18.4)	8.2 (5.3, 17.7)	0.206
IPSS	21 (13.5, 25.3)	19 (16, 22)	0.579
QOL score	6 (5, 6)	4 (4, 5)	0.004
Urinary peak flow rate (mL/s)	2.5 (0, 5)	5 (4, 8)	0.005
IIEF	7.5 (4, 18.5)	9 (1, 23)	0.937

Results were provided as median and (inter-quartile range)

The results of IPSS, QOL score, urinary peak flow rate, and IIEF in the study group were those before the onset of acute urinary retention

*PSA* Prostate specific antigen, *IPSS* International Prostate Symptom Score, *QOL* Quality of life, *IIEF* International Index of Erectile Function

### Study Endpoints

The primary endpoint was successful weaning of Foley catheter within 2 weeks of PAE. The secondary endpoints were safety outcome, clinical outcome, and urodynamic outcome at 1 month, and imaging outcome at 2 weeks.

### The Treatment

**Fig. 1:**
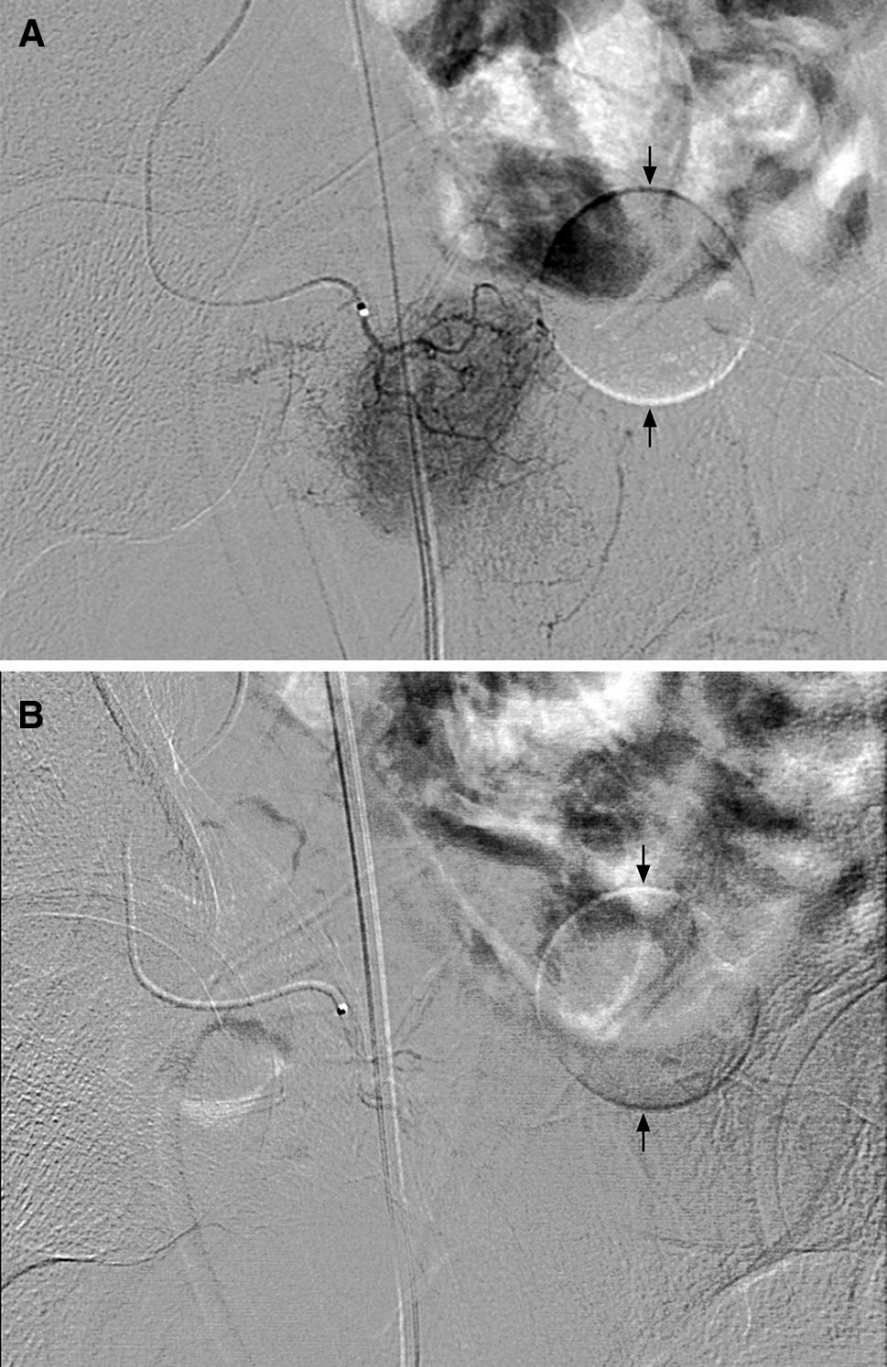
Selective arteriogram of the right prostate artery was performed at ipsilateral oblique 50° and caudal tilt 10° before embolization. Prostate vasculature was outlined. The prostate location was hinted with a Foley balloon that was pointed out with *arrows* (**A**). Selective arteriograms after embolization showed no contrast staining in the prostate (**B**)

**Fig. 2:**
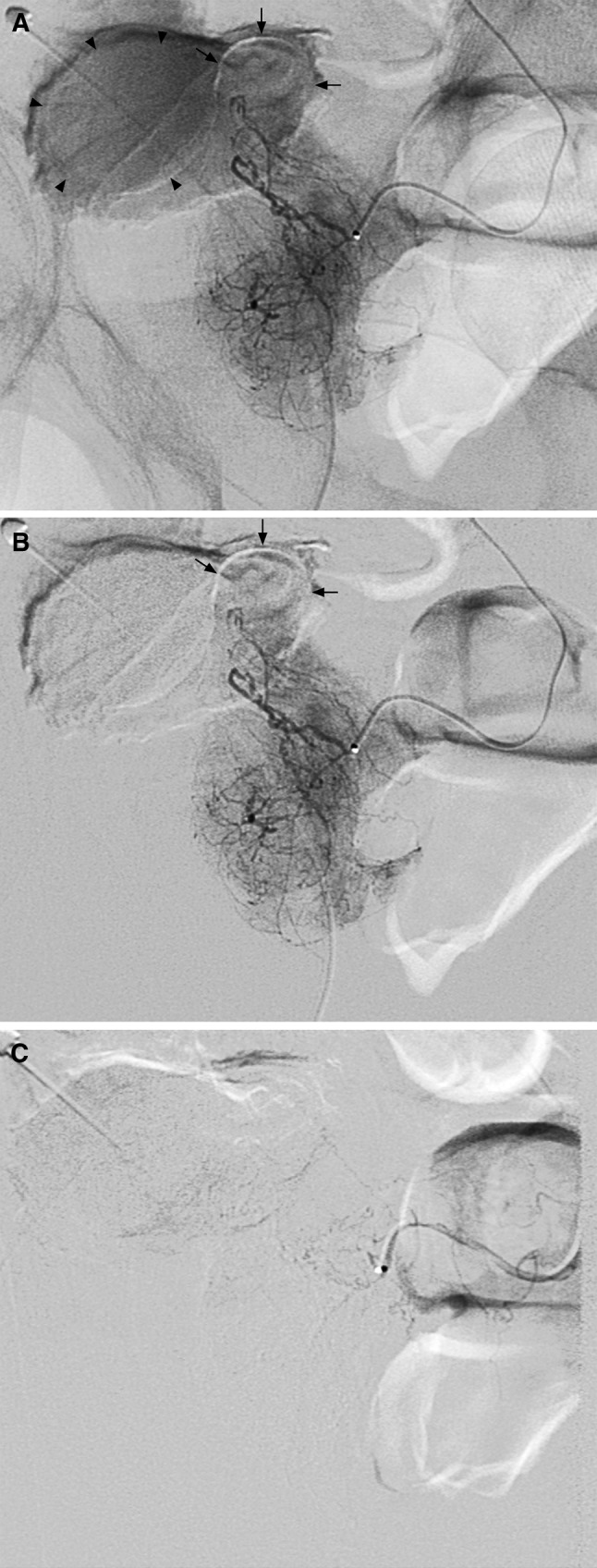
Selective arteriogram of the left prostate artery was performed at ipsilateral oblique 50° and caudal tilt 10° before embolization. Prostate vasculature was outlined between the urinary bladder on the *left* and the rectum on the *right* (**A**, **B**). The Foley balloon as pointed out with *arrowheads* located within the bladder lumen that was almost completely collapsed (**A**). The intravesical portion of the prostate as pointed out with *arrows* showed prostate vasculature inside (**A, B**). Selective arteriogram after embolization showed no contrast staining in the prostate (**C**)

PAE was performed with a standardized technique for all patients in both groups. This was an in-patient procedure. Pre-medication included the following: oral Voltaren SR100 mg (Diclofenac Sodium, Novartis Pharmaceuticals, Basel, Switzerland) once daily; famotidine 20 mg (Merck Sharp and Dohme. NSW, Australia) twice daily given for 2 days before the procedure, and in the morning of the procedure; Dulcolax rectal suppository 10 mg (Boehringer Ingelheim Pharma, Deutschland) given in the night before the procedure; and intravenous Ciprofloxacin 400 mg (Bayer HealthCare Ltd, Hong Kong) given within 1 h before the procedure. Patients were fasted for 6 h. For patients in the control group, a Foley catheter was placed for the procedure. In all patients, the Foley balloon was prepared before the procedure using 1 mL of contrast (Omnipaque 350, Amersham Health, Cork, Ireland) diluted to 10 mL (35 mgI/L) with water for injection and placed at the vesico-ureteric junction; this was used as a reference under fluoroscopy to localize the prostate. A single operator, who had 23 years of experience in endovascular procedures but no experience in PAE, performed all the procedures. The procedures were performed under local anesthesia using 5 mL of 1% lignocaine without parenteral sedation or analgesia. The right femoral artery was punctured using a Minipuncture Introducer Set (Cook Medical, Bloomington, USA). The arterial supply to the prostate was mapped with bilateral internal iliac angiography using tube rotation at ipsilateral 35°–50° and cranial-caudal angulation at 10°. Microcatheters (Merit Maestro 2.4F, Merit Medical Systems Inc., Utah, USA) were used for selective catheterization of the bilateral prostate arteries (Fig. 1). Cone-beam CT angiography or rotational angiography was performed to confirm catheter position and prostate staining when there was suspicion about potential non-prostate embolization. Embolization was performed using tris-acryl microspheres (Embosphere microspheres, Merit Medical) of diameter 100–300 μm, with 2 mL particle suspended in a mixture of 5 mL water and 10 mL Omnipaque 350, which was slowly delivered under fluoroscopic guidance until there was flow stasis in the prostate arteries (Fig. 2). Post-procedure medication included oral Voltaren SR100 mg once daily and famotidine 20 mg twice daily for 10 days, and oral ciprofloxacin 500 mg twice daily for 7 days. Alpha-blockers were discontinued 1 month after PAE.

### Outcome Assessment

The procedure time and fluoroscopy time as indicated in the angiographic equipment were captured. TWOC was performed on day 3 and 14 in the study group and on day 3 in the control group in the initial protocol, because the authors initially thought that it would take at least 3 days for the prostate to shrink and the obstructive effects to be relieved after embolization. Subsequently, it was observed that successful TWOC could be achieved on day 1. The protocol was therefore changed to have TWOC performed on day 1, 3, 7 in the study group and on day 1 after PAE. Regarding safety outcome, peri-PAE complications within 1 month were prospectively captured, including any puncture site complications, arterial dissection, intra-procedural or post-procedural pain in perineal, retropubic or urethral region; any other post-embolization syndrome such as nausea, vomiting, fever, or small amount of blood in urine or stool [6]; any signs and symptoms of prostatic or pelvic infection, infarction of the bladder, rectum, or genitals; or any event that had led to prolonged hospitalization or hospital readmission. For this purpose, patients were observed during the procedure and during the following 3 days of hospitalization and symptoms were prospectively captured, symptoms on day 2 and 3 were prospectively captured with telephone contact if the patient was discharged from hospital, and patients were interviewed at 1 month. For pain assessment, patients were asked to rate their pain severity from 0 (sensation of no pain) to 10 (the worst pain imaginable). Regarding imaging outcome, contrast-enhanced MRI was performed at baseline and at two weeks. Percent prostatic volume reduction from baseline and the percentage of non-perfused volume on contrast-enhanced MRI were estimated. The extent of non-perfused volume was used as an indicator to assess the status of prostate embolization. Regarding clinical outcome, the patients’ clinical symptoms were assessed with IPSS and Quality of Life score (QOL) at one month and compared with those at baseline. Regarding urodynamic outcome, urine peak flow rate, and post-void residual urine volume at 1 month were assessed and compared with those at baseline.

### Imaging Protocols

CTA was performed with a 64-slice multi-detector CT (General Electrics). The power settings were 100–120 kV and 200–300 mA; matrix, 512 × 512 pixels; field of view 360 × 360 mm; voxel size, 0.7 × 0.7 × 1.25 mm; collimation, 16 × 1.25 mm; and pitch, 1.3. Power injection settings were 100 mL of iodinated contrast (Omnipaque 350), injection rate 4 mL/s, and bolus triggering in the abdominal aorta. Threshold level for acquisition was 200 HU. The delay is usually 16–20 s. A 30-mL saline flush before and after contrast injection at the same rate as the contrast injection is performed in every patient. Sublingual vasodilator (0.5 mg nitroglycerin, Quilaban, Química Laboratorial Analítica Lda) was given before image acquisition to help identify small arteries. The mean acquisition time was 12 s for a scanning range of 30 cm.

MRI was acquired using a Philips Achieva Tx 3T scanner using a sense cardiac coil (receive) and a body coil (transmit). T2W multi-shot TSE pulse sequences were acquired in the sagittal, coronal, and axial planes. After the baseline scan, 0.2 mL/kg gadoteric acid (0.5 mmL/mL; DOTAREM, Guerbet, France) was injected intravenously by hand (2 mL/s) with 20 mL 0.9% saline flush. Starting from the time of injection, images were acquired at 6.9 s intervals with a total of 13 acquisitions. Post-contrast fat saturated e-THRIVE sequences were then performed in the axial and coronal planes. MRI data were processed with computer programs for volumetric assessment.

### Statistical Analysis

Continuous data were presented as median and inter-quartile range. Patient proportions in treatment outcome analysis were presented as percentages. Comparison of continuous variables between the two patient groups was performed with Mann–Whitney U test. Comparison of patient proportions between the two patient groups was performed with Fisher’s exact test. Difference between the two patient groups was considered not significant when *P* ≥ 0.05.

## Results

PAE with selective catheterization of bilateral prostate arteries was successfully performed in all 31 patients. All 31 patients who received PAE had completed imaging assessment at 2 weeks and clinical, urodynamics, and safety assessments at 1 month. The serum prostate-specific antigen (PSA) level was high at baseline; it was 14 μg/L [Median, inter-quartile range (IQR) 7.5, 18.4] and 8.2 μg/L (Median, IQR 5.3, 17.7) in the study group and control group, respectively (Table 2). There was no histology or MRI evidence of prostatic carcinoma in all 31 patients. The procedure time and fluoroscopy time in the study group were 116.5 min (Median, IQR 97.3, 144) and 34 min (Median, IQR 25.8, 43.8), respectively, and those in the control group were 116 min (Median, IQR 87, 162) and 31 min (Median, IQR 17, 36), respectively. There was no significant difference in the procedure time or fluoroscopy time between the two groups (Table 3). In the initial nine patients of the study group, TWOC was successful on day 3 in three patients and successful on day 14 in five patients; TWOC was failed in 1 patient. In the other seven patients of the study group, TWOC was successful on day 1 in two patients, day 3 in three patients, and day 7 in one patient; TWOC was failed in one patient. Altogether, 14 patients out of 16 in the study group had successfully weaned off Foley’s catheter within 2 weeks after PAE; the success rate was 87.5%. In the control group, TWOC was successful on day 3 in all the initial six patients, and on day 1 in all the other nine patients (Table 4). The two patients who failed TWOC after PAE underwent TURP, the symptoms of lower urinary tract obstruction due to BPH subsided afterward.

**Table 3:** Treatment outcome

Outcome parameters	Study group (*n* = 16)	Control group (*n* = 15)	*P* value
Weaned off Foley’s catheter	14 (87.5%)	15 (100%)	0.484
Total procedure time (min)	116.5 (97.3, 144)	116 (87, 162)	0.913
Fluoroscopy time (min)	34 (25.8, 43.8)	31 (17, 36)	0.247
Prostate volume at 2 weeks (mL)	65.6 (47.5, 90.4)	62.1 (34.8, 70.6)	0.239
Prostate volume reduction at 2 weeks (%)	12.4 (7.9, 17.4)	12.6 (4.3, 20.4)	0.663
Percent non-perfused volume at 2 weeks	79 (73, 81)	80 (74, 83)	0.389
Serum PSA level 1 month (μg/L)	5.2 (3.6, 7.4)	6.6 (3.4, 10.2)	0.678
Serum PSA reduction (%)	49 (34.9, 65.2)	37.1 (4.7, 58.4)	0.138
IPSS at 1 month	5.5 (5, 11.3)	7 (2, 11)	0.38
IPSS reduction ≥25% at 1 month	13 (92.9%)	14 (93.3%)	>0.999
IPSS reduction ≥50% at 1 month	10 (71.4%)	10 (66.7%)	>0.999
QOL at 1 months	2 (1, 3)	1 (0, 2)	0.223
QOL improved ≥1 at 1 month	14 (100%)	14 (93.3%)	>0.999
QOL improved ≥3 at 1 month	9 (64.3%)	8 (53.3%)	0.825
Peak flow rate at 1 month	8 (5, 10.1)	12 (9, 15)	0.051
Peak flow rate increase ≥2.5 mL/s at 1 month	11 (78.6%)	11 (73.3%)	>0.999
Peak flow rate increase ≥5 mL/s at 1 month	7 (50%)	8 (53.3%)	>0.999
IIEF at 1 months	4.5 (1.8, 19.8)	9 (3, 22)	0.605
IIEF reduction value	0 (0, 0)	0 (0, 0)	0.526

Continuous data of the results were provided as median and (inter-quartile range)

*PSA* Prostate specific antigen, *IPSS* International Prostate Symptom Score, *QOL* Quality of life score, *IIEF* International Index of Erectile Function

**Table 4:** Schedule and outcome of trial without catheter (TWOC)

Timing of TWOC (day)	Study group (*N* = 16)		Control group (*N* = 15)	
	*N* attempted	*N* succeeded	*N* attempted	*N* succeeded
1	(7)	(2)	(9)	(9)
3	9 + (5)	3 + (3)	6	6
7	(2)	(1)		
14	6	5		
Total *N* succeeded within 2 weeks		14		15

Number in brackets = *N* of patients in the second batch

Note that the total number of attempts of TWOC in the study group was greater than 16 since some of the attempts were unsuccessful and therefore repeated

There was no peri-procedural complications, no post-embolization pain of severity >2 out of 10, or any other adverse events. The results of other outcome assessments are shown in Table 3. The serum PSA level was substantially reduced at 1 month after PAE; it was 5.2 μg/L (Median, IQR 3.6, 7.4) and 6.6 μg/L (Median, IQR 3.4, 10.2) in the study group and control group, respectively. Prostate volume reduction ≥10% at 2 weeks occurred in 57.1% of patients in the study group and 53.3% of patients in the control group. Non-perfused prostate volumes were 79% (Median, IQR 73, 81) in the study group and 80% (Median, IQR 74, 83) in the control group. Reduction in IPSS ≥50% at 1 month occurred in ten patients (71.4%) of the study group and ten patients (66.7%) in the control group. Increase in QOL ≥3 at 1 month occurred in nine patients (64.3%) of the study group and eight patients (53.3%) of the control group. Increase in urine peak flow rate ≥5 mL/s at 1 month occurred in seven patients (50%) in the treatment group and eight patients (53.3%) in the control group. There was no significant difference between the two groups in all the outcome parameters.

## Discussion

This study showed that the success rate of PAE in relieving complete urinary obstruction in patients with AUR due to BPH is high (87.5%). There was no complication or sexual dysfunction after PAE. The treatment outcome as assessed by imaging, clinical, and urodynamic parameters had shown significant improvement as compared to baseline, and no difference between the groups with or without acute urinary retention. These findings have confirmed the finding of a previous study by Carnevale et al. [9], in which 11 patients with acute urinary retention caused by BPH treated with PAE resulted in successful catheter removal and symptomatic improvement in 91%. Patients can be treated safely with PAE, resulting in overall clinical improvement in lower urinary tract symptoms as assessed by QOL and urodynamic data.

These results implied that that PAE could be an effective and safe alternative to TURP for patients with AUR due to BPH, who fail to wean off urinary catheter despite having on alpha-blocker, are not amenable to general or regional anesthesia, or not willing to expose themselves to the risk of complications associated with TURP. Failure of PAE does not preclude the patient from receiving TURP.

The techniques of CTA, pelvic arteriogram, Foley balloon localization, and prostate artery catheterization used in this study have been reported before [9]. The use of a Foley balloon in PAE procedures for patients without AUR may not be justified anymore because it would induce patient distress and it carries a risk of urinary tract infection and urethral injury, given that the need for using a Foley balloon for anatomical localization of the prostate is diminished with the use of cone-beam CT or rotational angiography. Diclofenac was started 2 days before the procedure to reduce ischemic and inflammatory pain during the procedure. In the studies by Pisco et al., Naproxen 1000 mg twice daily was given for 2 days before PAE, and there was totally no pain during the procedure in 79% of patients [8, 10]. This may account for the absence of post-PAE pain of severity >2 out of 10 in all patients in our study, when post-PAE pain and other post-embolization syndrome were not uncommon in other studies. Microspheres of diameter 100–300 μm were used in this study, while microspheres of 300–500 μm were used in other studies [9, 13]. Although smaller sized microspheres could possibly be more effective in reaching the terminal branches of the prostate artery and causing more extensive ischemic necrosis of the prostate, they could be more risky in inadvertent embolization of non-target pelvic organs. Bladder wall necrosis presenting with severe intra-procedural pain had occurred after PAE in which polyvinyl alcohol particles of size 100–200 μm (Cook Medical) were used [10]. In a small-scale non-randomized comparative study with 15 patients in each arm, Embosphere microspheres of size 100–300 μm and size 300–500 μm were compared for use in PAE for BPH. Although there was no significant difference in functional and imaging outcomes between the two groups, there was a greater incidence of adverse events in the group of smaller particles [14]. In the current study, in which tris-acryl microspheres of size 100 to 300 μm were used, embolization-related complications did not occur, suggesting that it may be safe to use smaller sized microspheres. Regarding the determination of the optimal size of microspheres for PAE, further studies involving larger numbers of patients, longer durations of follow-up, clinical outcome, and imaging of the extent of necrosis would be necessary.

The high serum PSA level in both groups was probably due to BPH, as well as an element of prostatitis. The higher PSA level in the study group relative to the control group was likely due to a greater degree of prostatitis induced by the long-term indwelling intravesical catheter, which is not uncommon among this group of patients. Marked reduction in the PSA level after PAE was probably another indicator of the therapeutic effect of PAE on BPH, as well as resolution of prostatitis following the removal of the intravesical catheter.

The patient number of this study was relatively small, although the endpoints of procedure safety and effectiveness in weaning off the intravesical catheter within 2 weeks were clearly demonstrated. The follow-up period of this study was relatively short. Although substantial favorable changes in symptomatology, urodynamics, and serological parameters had already been shown at 1 month as compared to baseline, further changes at a later stage were not evaluated. A future report of longer term follow-up might be interesting. The study design of comparison to a control group allowed evaluation of the treatment effect of PAE in patients with AUR, with reference to those without AUR. It might be valuable to further evaluate the role of PAE in AUR in a randomized control trial of larger scale, in comparison with TURP, to study the complications, short-term and long-term outcomes of symptomatology and urodynamic changes, and the need and outcome of a repeat PAE.

In conclusion, PAE was probably safe and effective in weaning of catheter and relieving obstructive urinary symptoms in patients with AUR due to BPH, with treatment outcomes comparable to those without AUR. Further studies with longer follow-up and larger patient cohorts are needed.
